# TGF-β Signaling-Related Genes and Thoracic Aortic Aneurysms and Dissections

**DOI:** 10.3390/ijms19072125

**Published:** 2018-07-21

**Authors:** Norifumi Takeda, Hironori Hara, Takayuki Fujiwara, Tsubasa Kanaya, Sonoko Maemura, Issei Komuro

**Affiliations:** Department of Cardiovascular Medicine, The University of Tokyo Hospital, 7-3-1 Hongo, Bunkyo-ku, Tokyo 113-8655, Japan; hironorihara1022@gmail.com (H.H.); sasasatf5804@yahoo.co.jp (T.F.); sourmint1007@gmail.com (T.K.); irontennispart@yahoo.co.jp (S.M.); komuro-tky@umin.ac.jp (I.K.)

**Keywords:** Marfan syndrome, Loeys-Dietz syndrome, Shprintzen-Goldberg syndrome, fibrillin-1, endothelial dysfunction, angiotensin II, TGF-β paradox

## Abstract

Transforming growth factor-β (TGF)-β signaling plays a crucial role in the development and maintenance of various organs, including the vasculature. Accordingly, the mutations in TGF-β signaling pathway-related genes cause heritable disorders of the connective tissue, such as Marfan syndrome (MFS), Loeys-Dietz syndrome (LDS), and Shprintzen-Goldberg syndrome (SGS), and these syndromes may affect skeletal, ocular, pulmonary, and cardiovascular systems. Aortic root aneurysms are common problems that can result in aortic dissection or rupture, which is the leading cause of sudden death in the natural history of MFS and LDS, and recent improvements in surgical treatment have improved life expectancy. However, there is currently no genotype-specific medical treatment. Accumulating evidence suggest that not only structural weakness of connective tissue but also increased TGF-β signaling contributes to the complicated pathogenesis of aortic aneurysm formation, but a comprehensive understanding of governing molecular mechanisms remains lacking. Inhibition of angiotensin II receptor signaling and endothelial dysfunction have gained attention as a possible MFS treatment strategy, but interactions with TGF-β signaling remain elusive. Heterozygous loss-of-function mutations in TGF-β receptors 1 and 2 (*TGFBR1* and *TGFBR2*) cause LDS, but TGF-β signaling is activated in the aorta (referred to as the TGF-β paradox) by mechanisms yet to be elucidated. In this review, we present and discuss the current understanding of molecular mechanisms responsible for aortopathies of MFS and related disorders.

## 1. Introduction

Thoracic aortic aneurysms (TAAs) increase the risk of aortic dissection or rupture, and inherited forms of thoracic aortic aneurysms and dissections (TAADs) can be fatal early in life if patients do not receive appropriate care. Familial TAAD can be divided into two broad categories: syndromic (associated with abnormalities of other organ systems) and non-syndromic (with manifestations restricted to the aorta) [1,2]. Expression of disease-causing genes in syndromic TAAD is found in different tissues of the body, including the aorta, while expression of non-syndromic TAAD is restricted in vascular cells, such as smooth muscle cells (SMCs).

TGF-β signaling plays a crucial role in the development and maintenance of various organs, including the vasculature. Mutations in transforming growth factor-β (TGF-β) signaling pathway-related genes cause syndromic TAAD, such as Marfan syndrome (MFS), Loeys-Dietz syndrome (LDS), and Shprintzen-Goldberg syndrome (SGS), and these syndromes potentially affect skeletal, ocular, pulmonary, and cardiovascular systems [3,4]. Acute aortic dissection is the leading cause of sudden death in the natural history of MFS and LDS [5], and improved surgical management has increased life expectancy; however, comprehensive understanding of molecular mechanisms governing multiple aortic aneurysms and dissections remains unclear. Thus, there are no effective medical treatments to prevent aneurysmal formation [4]. In this review, we present and discuss current molecular knowledge of aortopathies caused by mutations in TGF-β signaling-related genes and related aortopathy models.

## 2. Marfan Syndrome

MFS is an autosomal dominant disorder of the connective tissue that affects cardiovascular (aortic aneurysm and dissection), skeletal (long limbs and fingers, scoliosis, and pectus deformities), ocular (ectopia lentis), and pulmonary (pneumothorax) systems [3]. Cystic medial necrosis (CMN) is a typical feature in the medial layer of the aortic aneurysmal wall, which is characterized by fragmentation and disorganization of elastic fibers, collagen production with fibrosis, accumulation of amorphous matrix components, and loss of nuclei [6]. Expression of SMC differentiation markers, such as αSMA, SM22α, and smoothelin, decrease in the medial layer [7], and inflammatory T lymphocytes and macrophages infiltrate aortic media and adventitia [8,9]. These data suggest that active signals contribute to the formation of the aortic aneurysm, and molecular mechanisms have been actively investigated since the causative *fibrillin-1* (*FBN1*) gene at chromosome 15q21 was identified by Harry Dietz and colleagues in 1991 [10]. Up to 97% of MFS patients who fulfilled the revised Ghent criteria for clinical diagnosis have *FBN1* mutations [11], a gene that encodes a major component of the extracellular matrix (ECM) microfibril, namely fibrillin-1 [10]. Fibrillin-1 is a large (350 kDa) glycoprotein that assembles to form 10–12 nm microfibrils in the ECM and can regulate TGF-β bioavailability by releasing latent TGF-β from the ECM in response to pathophysiological stimuli. Thus, mutated fibrillin-1 results in not only the structural weakness of connective tissue but also dysregulation of TGF-β signaling, both of which contribute to complicated pathogenesis in MFS.

### 2.1. Fibrillin-1 Regulates TGF-β Bioavailability

**Figure 1:**
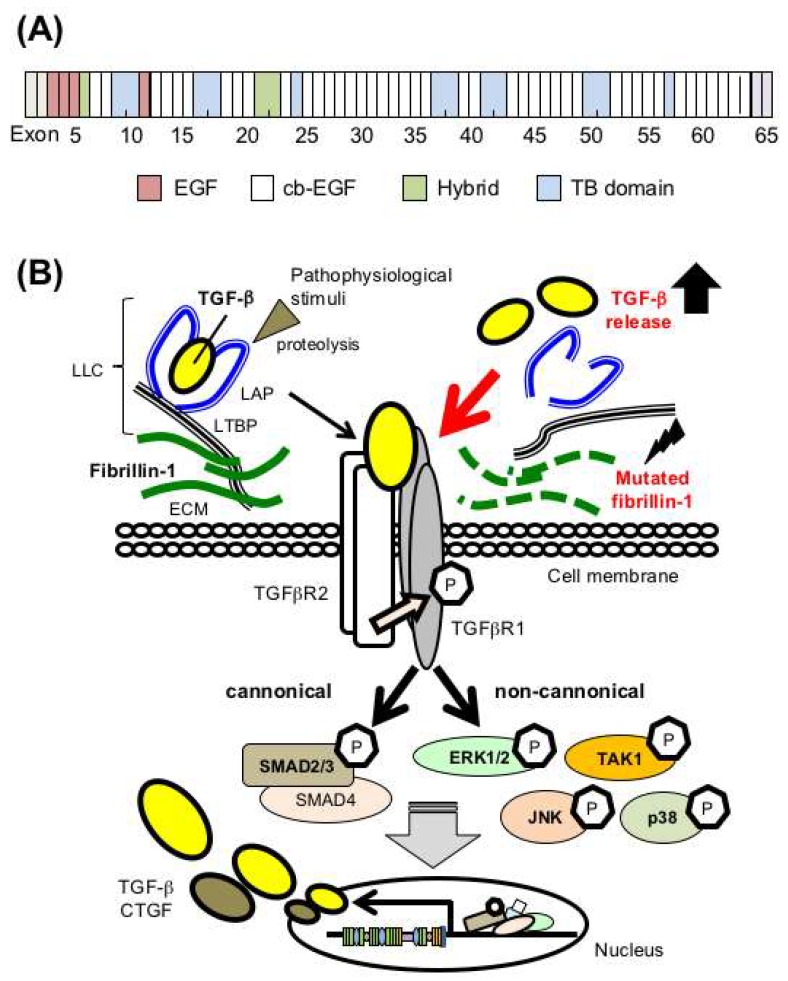
Mutations in the *FBN1* gene encoding fibrillin-1 cause dysregulation of TGF-β bioavailability in Marfan syndrome (MFS). (**A**) Domain structure of fibrillin-1. EGF, epidermal growth factor-like domains; cb-EGF, EGF-like domains with a calcium binding domain; TB, TGF-β binding protein-like domains; Hybrid, hybrid domain containing features of both TB and cb-EGF domains. (**B**) (left side) TGF-β is secreted in an inactivated latent form that requires proteolysis for activation. (right side) Mutated fibrillin-1 in MFS leads to failed sequestration of latent TGF-β in the ECM and subsequent activation of canonical and noncanonical TGF-β signaling cascades, which would play critical roles in MFS pathogenesis. ‘P’ indicates phosphorylation. LLC, large latent complexes; LAP, latency-associated peptide; LTBP, latent TGF-β binding protein; CTGF, connective tissue growth factor.

The *FBN1* gene contains 65 exons, and the encoded microfibrillar protein fibrillin-1 contains 47 epidermal growth factor (EGF)-like domains and seven TGF-β binding protein-like (TB) domains, which are characterized by six and eight conserved cysteine residues that form three and four intra-module disulfide bonds, respectively (Figure 1A). Of the 47 EGF domains, 43 contain a consensus sequence for calcium binding (cb-EGF), which play crucial roles in microfibril stability and assembly [3,12,13,14]. 

Fibrillin-1 and microfibrils regulate the bioavailability and local activity of TGF-β; TGF-β cytokines are generally secreted in an inactive form as a large latent complex (LLC) that contain the cytokine, latency-associated peptide (LAP), and latent TGF-β binding protein (LTBP) anchored to the ECM with fibrillin-1. Normally, inflammatory proteolytic enzymes such as elastase and/or certain physiological stimuli lead to microfibril degradation, which allows the release of diffusible active TGF-β that could act as a central regulator of the pathophysiological response, upregulating the expression of TGF-β, connective tissue growth factor (CTGF), and ECM [15]. In contrast, reduced or abnormal fibrillin-1 in MFS leads to failed sequestration of TGF-β, and the ensuing overactivity of TGF-β signaling cascades play crucial roles in MFS pathogenesis [16] (Figure 1B). Total plasma TGF-β1 levels were elevated in MFS patients [17], and β-blockers and angiotensin II receptor blocker (ARB) losartan, which are the current gold standards for MFS treatment, reduce plasma TGF-β1 concentrations [17].

### 2.2. Genotype-Phenotype Relationships in Marfan Syndrome

**Figure 2:**
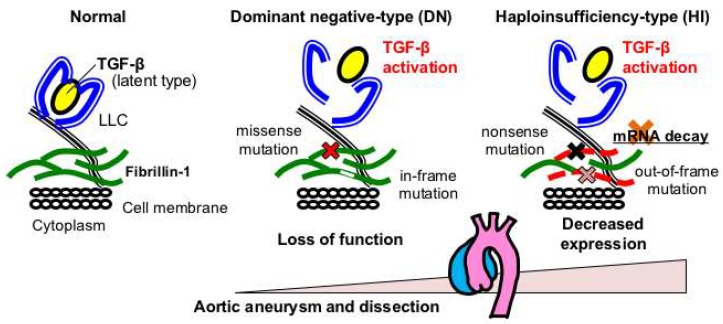
*FBN1* genotypes and aortopathy in MFS. Haploinsufficient (HI)-type variants, such as nonsense and out-of-frame variants that presumably cause nonsense-mediated mRNA decay (NMD), result in more severe aortic phenotypes than those with dominant negative (DN)-type variants, such as missense and in-frame variants that are expected to exert loss-of-function effects.

More than 3000 pathogenic mutations have been identified in *FBN1* and are distributed throughout the entire length of the gene. The relationships between *FBN1* genotypes and phenotypes have been extensively reported [18,19,20,21,22,23,24,25,26]. For example, a higher probability of ectopia lentis is found with a missense mutation substituting or producing a cysteine residue [21]. In addition, exons 24–32 are recognized as a critical region for the neonatal form of MFS [18], which is characterized by severe mitral and/or tricuspid valvular insufficiency and pulmonary emphysema [19]. Faivre et al. reported that mutations in exons 24–32 define a high-risk group for cardiovascular manifestations at all ages [20]. Furthermore, we and others have demonstrated that patients with haploinsufficient (HI)-type *FBN1* variants, such as nonsense and out-of-frame variants that presumably cause nonsense-mediated mRNA decay (NMD), have more severe aortic phenotypes than those with dominant negative (DN)-type mutations, such as missense and in-frame variants that are expected to exert loss-of-function effects [21,22,23,24,25,26] (Figure 2). Very recently, we have identified deleterious variants among DN patients, showing that patients with mutations affecting or creating cysteine residues and in-frame deletion variants in the cb-EGF domains of exons 25–36 and 43–49 (DN-CD variants) had a 6.3-fold higher risk for aortic events than DN-nonCD patients, which is comparable to or more deleterious than HI variants [26].

The underlying mechanisms in the relationship between genotype and aortopathy may not be as simple as the classification of genotype because variability in phenotypes has been reported not only across families with the same *FBN1* genotype but also within families [26]. The pathogenic variant *per se* may not be the only determinant of phenotype severity. However, previous studies have suggested mechanisms that implicate *FBN1* mutations in pathogenesis. For example, variants associated with neonatal and/or severe forms of MFS cluster in a center region of fibrillin-1 (exons 24–32) [18,19,20]. Exons 44–49-encoded sequence can enhance endogenous active TGF-β1 and SMAD2 signaling [27]. Cysteine substitutions in the cb-EGF domains disrupt one of the 3 disulfide bonds that play an important role in protein structure stabilization [28], and calcium binding to cb-EGF modules plays a crucial role in microfibril stability and assembly [29]. The complete loss of one *FBN1* allele can lead to aortic root dilatation with high penetrance [30]. The relationships among genotypes, changes in signals, and phenotypes remain elusive and warrant further investigation.

### 2.3. Murine Model of Marfan Syndrome

To elucidate the molecular mechanisms leading to MFS, two genetically modified *Fbn1* murine models have been widely studied [3]: hypomorphic mgR/mgR mice [31] and heterozygous knock-in *Fbn1^C1039G/+^* mice [32,33] recapitulate degradation of abnormal fibrillin-1 and the dominant-negative effect, respectively. mgR/mgR mice expressing only ~20% of fibrillin-1 protein rapidly develop ascending aortic aneurysms with macrophage infiltration, intimal hyperplasia, elastic fiber fragmentation, calcified media [31], and proteoglycan accumulation, such as aggrecan and versican [34]. In addition, they display MFS-like features in skeletal and pulmonary systems, such as severe kyphosis, overgrowth of ribs, and air space dilatation, with destructive changes and peribronchiolar inflammation. mgR/mgR mice die within the first 6–9 months of life because of aortic disorders and the pulmonary insufficiencies associated with severe kyphosis.

The *Fbn1^C1039G/+^* knock-in mice were generated via substitution of a cysteine with a glycine at amino acid 1039 in exon 25 of the mouse *Fbn1* gene [32,33,35]. Homozygous knock-in mice (*Fbn1^C1039G/C1039G^*) die from aortic dissections in the perinatal period, whereas heterozygous *Fbn1^C1039G/+^* mice have relatively long-term survival of more than 90% at 8 months of age [36]. Moreover, after two months of age, *Fbn1^C1039G/+^* mice gradually recapitulate aspects of skeletal, pulmonary, and cardiovascular disorders. In the aorta, elastic fiber fragmentation, disarray of SMCs, and excessive collagen and proteoglycan deposition deteriorate progressively, with occasional elastic fiber calcification.

### 2.4. Canonical and Noncanonical TGF-β Signaling in Marfan Syndrme

**Figure 3:**
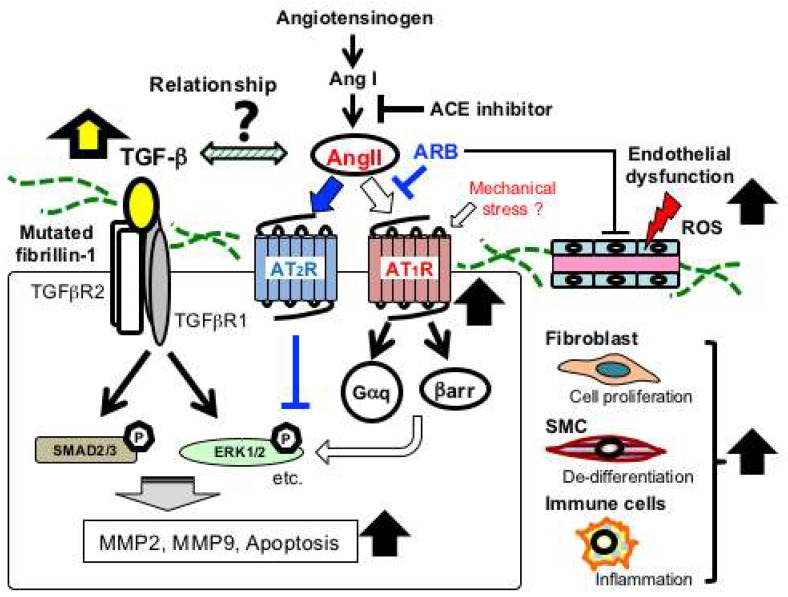
Schematic of major signaling pathways implicated in aortic aneurysmal formation in MFS. *FBN1* mutations have been considered to mainly cause impaired smooth muscle cells (SMC) with increased activity of matrix metalloproteinases (MMP-2 and MMP-9) and proapoptotic factors. Recent studies have demonstrated that multiple signaling pathways are activated in various vascular-composing cells, including endothelial cells, fibroblasts, and immune cells. Angiotensin II (AngII) type 1 receptor blocker (ARB), such as losartan, has been proven effective in preventing noncanonical ERK1/2 activation and aneurysmal formation (blue lines and arrows). Endothelial dysfunction and excessive reactive oxygen species (ROS) production also play crucial roles in the aneurysmal progression. ACE, angiotensin-converting enzyme; AT_1_R, Ang II type 1 receptor; AT_2_R, AngII type 2 receptor; βarr, β-arrestin; Gαq, a G-protein α-subunit; MMP, matrix metalloproteinase.

Aortic TGF-β1 expression and total plasma TGF-β1 levels are elevated in *Fbn1^C1039G/+^* and mgR/mgR mice. The SMAD-dependent canonical TGF-β signaling is activated in affected tissues, as indicated by increased accumulation of phosphorylated SMAD2 (pSMAD2), and thus systemic administration of TGF-β neutralizing antibody (NAb) can prevent some disease manifestations, including the aortic dilatation [16,32,35] (Figure 3).

In addition, recent reports demonstrated that SMAD-independent noncanonical pathways are also activated, and the activation of extracellular signal-regulated kinases (ERK1/2) serves as a prominent driver of aortic aneurysm formation [36] (Figure 3). *Fbn1^C1039G/+^* mice show significant increase in activation of ERK1/2 and mitogen-activated protein kinase kinase 1 (MEK1), which is the upstream activator of ERK1/2. Signals are inhibited by systemic TGF-β NAb treatment, and the selective MEK1/2 inhibitor RDEA119 (refametinib) ameliorates aortic growth, whereas SMAD2 and other noncanonical signals, such as Jun N-terminal kinase (JNK1) and p38, are unchanged [36]. These data suggested that TGF-β-driven ERK1/2 activation contributes to aneurysmal formation in MFS and that antagonism of this pathway may be therapeutically useful, but further studies are also necessary to examine the involvement of other noncanonical signaling components, such as TRAF6-TAK1 pathway [37,38], in various disease states. 

### 2.5. Angiotensin II Receptor Signaling in MFS

Although relationships between TGF-β and AT_1_R signaling pathways in MFS remain elusive, the blockade of Angiotensin II (AngII) type 1 receptor (AT_1_R) signals has been proven effective in preventing ERK1/2 activation and aneurysmal progression (Figure 3). AngII is the principal effector hormone in the renin-angiotensin system (RAS) that regulates blood pressure and fluid balance and also exerts a pro-inflammatory cytokine that promotes cell proliferation, inflammation, and fibrosis [39]. Habashi et al. reported that AT_1_R blocker (ARB) losartan reduced blood pressure and inhibited aortic aneurysm formation in *Fbn1^C1039G/+^* mice, but it could not in *Fbn1^C1039G/+^* mice lacking the AngII type 2 receptor gene (*Fbn1^C1039G/+^*; *AT2^−/−^*). In addition, the angiotensin-converting enzyme (ACE) inhibitor enalapril, which blocks AngII formation, did not adequately inhibit aneurysm formation, regardless of the same antihypertensive effect as losartan [40]. Furthermore, Cavanaugh et al. reported that subpressor doses of AngII could accelerate aneurysmal formation and dissection in *Fbn1^C1039G/+^* mice [41]. These observations suggested that losartan may exert favorable effects via activating or preserving AT_2_R signal cascades. Accordingly, selective AT1-receptor blockade with protective nature of AT_2_R signaling is an ideal therapeutic option for MFS patients, and recent clinical trials have shown that the inhibitory effect of losartan on the growth of aortic aneurysms is equivalent to β-blockers [4,42,43,44]. However, the overwhelming favorable effect has not been achieved.

The mechanisms by which the RAS system promotes aneurysmal formation have been actively investigated [45,46,47]. The selective non-peptide AT_2_R agonist compound 21 (C21) alone did not effectively ameliorate aneurysmal formation in *Fbn1^C1039G/+^* mice [45], and thus the selective blockade of AT_1_R downstream could be a preventive and/or therapeutic tool. AT_1_R is a G-protein-coupled receptor (GPCR) and activated by Gαq-protein-dependent and β-arrestin-dependent (G-protein independent) mechanisms. β-arrestin 2 is a multifunctional scaffold protein that binds to phosphorylated GPCRs such as AT_1_R, and regulates numerous signaling pathways, including proproliferative and profibrotic signals in aortic SMCs [46]. When *Fbn1^C1039G/+^* mice were crossed with β-arrestin 2 knockout mice, *Fbn1^C1039G/+^*:βarr2^−/−^ mice displayed slower aortic dilatation, in which AT_1_R-mediated ERK1/2 activation were decreased [46], suggesting that AT_1_R/β-arrestin-biased ligands may offer a new class of therapeutic agents for treatment. In addition, AT_1_R signaling in the vascular endothelium has recently been reported to play important roles in aneurysmal formation [47]. Galatioto et al. generated endothelial cell- and SMC-specific AngII type 1a receptor (*At1ar*) gene disruption in mgR/mgR mice by using *Cdh5-Cre* and *SM22-Cre* transgenic mice, respectively, and fibrillin-1 hypomorphic:*Agt1ar^Cdh5−/−^* mice revealed increased median survival associated with mitigated aneurysm growth and media degeneration and reduced levels of pERK1/2, but not pSMAD2 [47]. By contrast, the fibrillin-1 hypomorphic:*Agt1ar^Sm22−/−^* mice did not show apparent changes in TAA pathology despite normalized pERK1/2 and pSMAD2 levels. These data indicate that physiological AT_1_R signaling in the intimal and medial layers has distinct regulatory roles in aortic homeostasis and function, and improper AT_1_R signaling in the vascular endothelium may be a significant determinant of aneurysmal formation.

### 2.6. Endothelial Dysfunction in MFS

In MFS patients, endothelial dysfunction and increased aortic stiffness are associated with aortic aneurysmal formation [48,49,50], and losartan attenuates impairment of endothelial function in MFS mice and patients (Figure 3). Sellers et al. reported that *Fbn1^C1039G/+^* mice crossed with hypotensive *Agt1ar* knockout mice (*Fbn1^C1039G/+^*:*Agt1ar*^−/−^) showed unabated aortic aneurysmal formation [51]. However, losartan treatment still had a favorable effect on disease progression, suggesting that losartan’s anti-remodeling properties might be independent of its blood-lowering effect [51]. In addition, losartan could increase endothelial nitric oxide (NO) release, and it did not ameliorate aneurysmal formation in *Fbn1^C1039G/+^* mice treated with L-NAME (N omega-nitro-L-arginine methyl ester), a nitric oxide synthase (NOS) inhibitor. Furthermore, *Fbn1^C1039G/+^* mice expressing a constitutively active endothelial NOS mutation [eNOS 1176 Ser-to-Asp knock-in (S1176D)] showed attenuated aortic disease, whereas *Fbn1^C1039G/+^* mice expressing an inactive form of an eNOS mutation [eNOS 1176 Ser-to-Ala knock-in (S176A)] caused a nonsignificant increase in the aortic diameter [51]. These data might indicate that losartan’s effect on aortic aneurysmal formation is NO-dependent, and protection of endothelial function might be of therapeutic significance to prevent aortic aneurysmal formation.

### 2.7. Oxidative Stress in MFS

Excessive amount of reactive oxygen species (ROS), termed oxidative stress, impairs endothelial function, and mediates the progression of various vascular disorders, including MFS. Various mechanisms and enzymes are involved in ROS production, such as NADPH oxidases (NOXes), xanthine oxidase (XO), and nitric oxidase synthase (NOS). Normal levels of ROS regulate vascular tone, proliferation, and cell signaling. Fiorillo et al. reported that plasma levels of protein carbonyl content (protein CO), accounting for ROS attacks on proteins, was significantly higher in MFS patients [52]. Yang et al. reported that 8-isoprostane, a biomarker of oxidative stress, was increased in the plasma and aortic homogenates of *Fbn1^C1039G/+^* mice, and superoxide-producing enzymes, such as NOX2, XO, and inducible NOS (iNOS/NOS2), increased in the aorta, while antioxidant enzymes, such as superoxide dismutases (SOD1 and SOD2), were decreased [53]. In addition, Jimenez-Altayo et al. reported that, among NOXes present in vascular cells, including the hydrogen peroxide (H_2_O_2_)-generating enzyme NOX4 and the superoxide-generating enzymes NOX1, NOX2, and NOX5, NOX4 was especially increased in the dilated zone of *Fbn1^C1039G/+^* mice aorta, and *Fbn1^C1039G/+^* mice crossed with *Nox4* knockout mice (*Fbn1^C1039G/+^*:*Nox4^−/−^*) had smaller aortic diameters [54]. NOX4 constitutively produces low amounts of cellular ROS (H_2_O_2_), contributing to physiological responses, but expression is significantly augmented by TGF-β and in vascular injury, which might contribute to the progression of aneurysmal formation.

Furthermore, Oller et al. reported that *Fbn1^C1039G/+^* mice had markedly elevated levels of iNOS/NOS2 and NO production; however, eNOS/NOS3 levels essential for keeping baseline vascular tone, were unaffected. *Fbn1^C1039G/+^* mice crossed with *Nos2* knockout mice (*Fbn1^C1039G/+^*:*Nos2^−/−^*) had smaller aortic diameters [55]. The data might suggest that excessive NO generation by NOS2 also plays a crucial role in aortic aneurysmal formation, presumably because excessive oxidative stress reduces bioavailability of NO through direct inactivation via production of peroxynitrite (ONOO^−^). This leads to further ROS production (NOS uncoupling).

Preferable effects of drugs with antioxidant activity on aortic aneurysmal formation have been reported: losartan, a HMG-CoA reductase inhibitor (statin) pravastatin [56], and resveratrol found in red wine [57]. The prevention and treatment of lifestyle-related diseases, including hypertension, dyslipidemia (hyperlipidemia), and diabetes, may be a desirable approach to prevent multiple aortic aneurysms and dissections, given these conditions can increase ROS production and may trigger more serious cardiovascular disease in middle-aged and elderly patients undergoing aortic root replacement (ARR). Van den Doncket et al. reported that *Fbn1^C1039G/+^* mice crossed with atherosclerosis-prone *ApoE* deficient mice (*ApoE^−/−^*:*Fbn1^C1039G/+^*) died suddenly, and elastin fragmentation led to intraplaque neovascularization, plaque rupture, myocardial infarction, and stroke in *ApoE^−/−^*:*Fbn1^C1039G/+^* mice [58].

### 2.8. Alterations in Other Signal Transduction Pathways and Biomarkers

Other pivotal transduction pathways are altered primarily and/or secondarily in the aorta [3]. In mgR/mgR mice, expression levels of pro-inflammatory cytokines, such as interleukin-6 (IL-6), monocyte chemoattractant protein-1 (MCP-1/CCL2), and granulocyte/macrophage-colony stimulating factor (GM-CSF), increased, and *IL-6* depletion ameliorated progressive aortic elastin degradation and aneurysmal formation [59]. In addition, in association with degradation of elastin and apoptotic cell death of the medial layer, activity of matrix metalloproteinases (MMP-2 and MMP-9) and proapoptotic factors (Bax and cleaved caspase-3, caspase-9) increased in the aortic walls [7] [33,60,61,62] (Figure 3).

Many relevant micro-RNAs (miRs) have been reported to play important roles in aortic aneurysm formation [63,64,65], and miR-29b is crucial for the early development of aortic root/ascending aneurysm in *Fbn1^C1039G/+^* mice [66,67]. The miR-29 family (miR-29a, miR-29b, and miR-29c) are enriched in fibroblasts and directly target at least 16 ECM genes, such as collagen isoforms (COL1A1, COL1A2, and COL3A1), fibrillin-1 (FBN1), and elastin (ELN), in several organs [63]. Merk et al. reported that miR-29b expression was upregulated in the ascending aorta of *Fbn1^C1039G/+^* mice beginning at 2 weeks, then peaked by 4 weeks, and returned to baseline by 8 weeks [66]. A miR-29b blockade administered intravenously and retro-orbitally prevented early aneurysmal development, which was closely associated with an increase in elastin production and stability and a decrease in MMP activity. Inhibition of aneurysmal formation via TGF-β neutralizing antibody (NAb), losartan, or resveratrol treatment was also accompanied by decreased levels of miR-29b in *Fbn1^C1039G/+^* mice [57,66]. These data suggest that miR-29b suppression could be a potential therapeutic target for reducing aneurysmal formation in MFS.

### 2.9. Beneficial Roles of TGF-β Signaling during Early Aortic Development

Increasing experimental evidence highlights the beneficial roles of TGF-β signaling during the early developmental period in MFS mice. In particular, TGF-β NAb treatment from postnatal day 45 (P45) ameliorated aneurysmal formation, but early initiation from P16 exacerbated it in mgR/mgR mice [68]. In addition, when *Fbn1^C1039G/+^* mice were crossed with *Smad4* or *Tgfb2* heterozygous knockout mice for the purpose of attenuating TGF-β signaling, *Fbn1^C1039G/+^*:*Smad4^+/−^* mice died prematurely due to proximal aortic rupture [36]. *Fbn1^C1039G/+^*:*Tgfb2^+/−^* mice rapidly developed aortic aneurysms [69]. These data suggest protective roles of TGF-β signaling during early postnatal aortic development in MFS. Recent reports also showed that basal levels of TGF-β signaling in SMCs impede aortic dilatation in normal aorta [70,71], even after adolescence, which is discussed in later sections on LDS and related genetic murine models. This insight may shed light on the significance of TGF-β signaling on aortopathy for each disease and genetic model.

## 3. Loeys-Dietz Syndrome and Related Genetic Murine Models

LDS is a recently identified MFS-like syndrome, characterized by a triad of arterial tortuosity and aneurysm, widely spaced eyes (hypertelorism), and bifid uvula. Rapidly progressive aortic/arterial tortuosity and aneurysm result in ruptures at an early age and at smaller dimensions, compared to MFS [72,73]. The pathogenic mutations in genes encoding TGF-β receptors 1 and 2 (*TGFBR1* and *TGFBR2*, respectively) were first identified as causes of LDS in 2004–2005 [74,75]. Until recently, mutations in *SMAD3* [76,77], *TGFB2* [69,78], and *TGFB3* [79], which encode members of the TGF-β/SMAD signal transduction pathway, were also reportedly associated with diseases that resemble MFS. Thus, the revised nosology for LDS diagnosis classified such patients according to the mutated genes: *TGFBR1* (LDS 1), *TGFBR2* (LDS 2), *SMAD3* (LDS 3), TGFB2 (LDS 4), and TGFB3 (LDS 5) [73].

### 3.1. Heterozygous Loss-of-Function Mutations in TGFBRs Cause Loeys-Dietz Syndrome

TGFBR1 and TGFBR2 are transmembrane serine/threonine kinase (STK) receptors comprising 9 and 7 exons, respectively. TGF-β ligands bind to TGFBR2, inducing its dimerization, and enables the TGFBR2 homodimer to form a stable hetero-tetrameric complex with the TGFBR1 homodimer, which leads to the subsequent activation of SMAD2 and SMAD3 (canonical SMAD-dependent pathway). In addition, TGFBR1 activates SMAD-independent (noncanonical) signaling mediated by TRAF6, TAK1, and the p38 MAPK/JNK pathway [80]. These TGF-β signaling pathways are crucially involved in the development and maintenance of various tissues, including vessels and craniofacial growth and patterning. TGFBR3 plays a crucial role in regulating embryonic development [81], and often functions as a co-receptor with other TGF-β receptor superfamily members. Human LDS phenotypes, such as cleft palate and calvaria defects, were observed in neural crest-specific *Tgfbr1* or *Tgfbr2* knockout mice [82,83,84], and activation of TGFBR1/TGFBR3-mediated, noncanonical, TRAF6/TAK1/p38 signaling pathway is reported to be responsible for the craniofacial malformations [84], however, the in vivo involvement of TGFBR3 signaling in LDS aneurysmal formation, has not been reported.

Most pathogenic variants of *TGFBR1* and *TGFBR2* in LDS are missense substitutions of evolutionarily conserved residues within the STK domains that have been verified in vitro and/or predicted to be associated with loss-of-function [85,86]. However, chronic consequences of such heterozygous variants reportedly lead to an increased accumulation of phosphorylated SMAD2 (pSMAD2) in the aortic wall [72,75], suggesting the paradoxical upregulation of TGF-β signaling in vivo [87]. Consistently, knock-in mice with loss-of-function mutations (*Tgfbr1^M318R/+^* and *Tgfbr2^G357W/+^*) recapitulated vascular, craniofacial, and skeletal manifestations of LDS. TGF-β signaling was upregulated in the aorta of *Tgfbr1^M318R/+^* and *Tgfbr2^G357W/+^* mice, in which profuse CD45^+^ inflammatory cells infiltrated in thickened medial and adventitial layers, and *Tgfb1* expression increased [88]. This suggested that loss-of-function missense mutations of one allele of either TGF-β receptor gene is sufficient to cause LDS, but TGF-β signaling is activated in the aorta by mechanisms yet to be elucidated.

### 3.2. Haploinsufficiency-Type Mutations in TGFBRs do not Cause Loeys-Dietz Syndrome

Knock-in mice with loss-of-function mutations manifest aspects of LDS, whereas heterozygous knockout mice (*Tgfbr1^+/−^* and *Tgfbr2^+/−^*) do not develop any phenotypes [88]. Goudie et al. reported familial cases with primary multiple self-healing squamous epithelioma (MSSE) of the skin, caused by truncating mutations in the STK domain of the *TGFBR1* gene without LDS-like phenotypes [89,90]. DN-type and HI-type mutations in the STK domain induce LDS and MSSE, respectively [90], and we have recently observed pathology in LDS patients that may support this proposed but not yet validated mechanism [91]. Fujiwara et al. focused on a novel splice donor site variant in the *TGFBR1* gene (IVS5 + 1G > A) causing a familial case of LDS without MSSE phenotypes, which was predicted to mediate in-frame exon 5 skipping within the STK domain. A similar variant is also expected to cause the in-frame deletion of exon 5 (IVS4-2A > C) and reported to induce MSSE without LDS-like phenotypes [91]. Analysis of in vivo RNA transcription products and ex vivo functional splicing assays revealed that the IVS5 + 1G > A variant produced two in-frame transcripts as a result of exon skipping and cryptic donor splice-site activation. The IVS4-1A > C variant generated an out-of-frame transcript due to cryptic acceptor splice-site activation. Our results strongly supported that missense and truncating variants in the STK domain induce LDS and MSSE, presumably through the DN and HI effect, respectively [91].

### 3.3. Inactivation of Both Tgfbr2 Alleles Causes Aortic Aneurysm in Mice

To resolve the mechanism of paradoxical TGF-β signaling activation and clarify the roles of TGF-β signaling in the aortas of LDS patients, the stoichiometry of TGF-β receptor complexes, composed of two *Tgfbr1I* and two *Tgfbr2* subunits, warrants consideration, despite the presence of the heterozygous loss-of-function mutations. As described before, heterozygous knockout mice (*Tgfbr1^+/−^* and *Tgfbr2^+/−^*) do not develop any phenotypes [88]. In contrast, Li et al. reported that mice with both *Tgfbr2* alleles in SMCs postnatally disrupted (*Myh11-CreER^T2^*;*Tgfbr2^fl/fl^*) developed aortic aneurysms [70]. However, pSMAD2 levels in the aorta decreased, unlike in LDS, and expression of SMAD2, TGF-β ligands, and MAPK activation (p-p38 and p-ERK1/2) significantly increased in the aorta. Hu et al. also reported that two postnatal SMC-specific *Tgfbr2* knockout mice (*Acta2-CreER^T2^*;*Tgfbr2^fl/fl^* and *Myh11-CreER^T2^*;*Tgfbr2^fl/fl^*) developed severe aortopathy with hemorrhage, ulceration, dissection, accumulation of macrophage markers, elastolysis, abnormal proteoglycan accumulation, and aberrant SMC gene expression [71]. These data suggest that basal levels of TGF-β signaling in SMCs can impede aortic dilatation, presumably by promoting postnatal aortic wall homeostasis, and the ablation of TGF-β receptor signaling may cause aortic aneurysm via other mechanisms different from LDS. On the other hand, there is consistent activation downstream of the TGF-β receptor in LDS aortic aneurysms.

### 3.4. A Speculated Mechanism of TGF-β Paradox in Loeys-Dietz Syndrome

**Figure 4:**
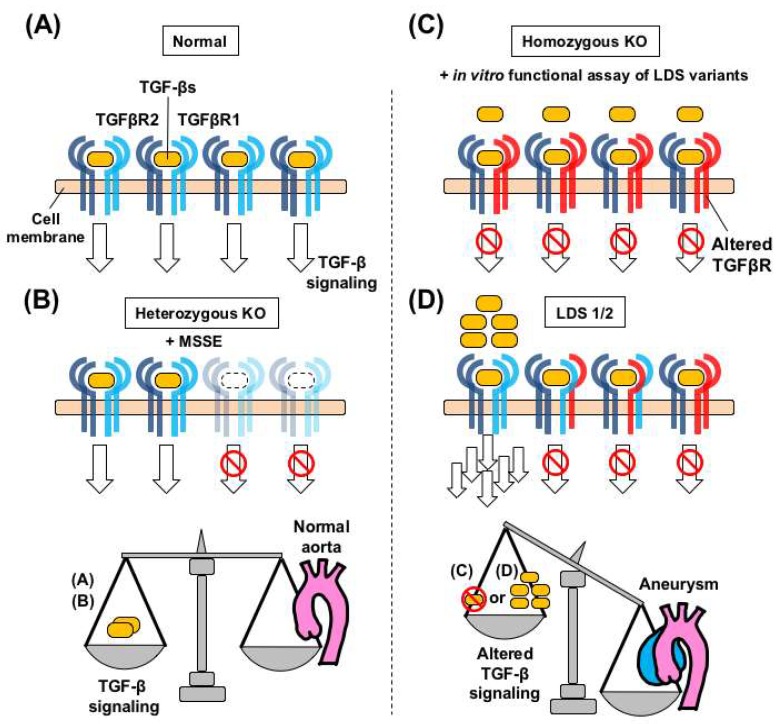
Dysregulated TGF-β signaling and aortopathy in Loeys-Dietz syndrome (LDS) and related genetic models. (**A**) Each TGFBR1 and TGFBR2 STK acts as a homodimer on the cell membrane in a normal aorta. (**B**) Basal levels of TGF-β signaling, through the wild-type/wild-type receptors, are maintained, in heterozygous *Tgfbr1* and *Tgfbr2* knock-out (KO) mice. This scheme holds true in patients with MSSE carrying truncating pathogenic variants in the STK domain of TGFBR1, who do not develop aortic aneurysms [91]. (**C**) Postnatal homozygous TGF-β receptor knockout mice, such as *Myh11-CreERT2.Tgfbr2^f/f^* [70], develop aortic aneurysms without activation of intracellular TGF-β signaling. This signaling landscape holds true in in vitro functional assays of LDS variants. (**D**) In LDS patients with loss-of-function variants, over-secreted TGF-β can be transmitted through homodimers of the remaining wild-type TGFBR homodimer complex. Dysregulated TGF-β signaling in the aortic wall is associated with aneurysmal formation, but the molecular processes leading to such aneurysms may be different, depending on the genetic conditions and animal models used.

Dysregulated TGF-β signaling in the aortic wall could lead to the development of aortic aneurysms, but the molecular processes that drive the initiation and/or progression of aortic aneurysms might be different, depending on the genetic conditions and animal models used (Figure 4). Considering the stoichiometry of TGF-β receptor complexes in heterozygous knockout mice (*Tgfbr1^+/−^* and *Tgfbr2^+/−^*) and MSSE patients with HI-type mutations, the existing TGF-β receptor complexes are all intact, and expression levels are theoretically one-half of the wild-type phenotype. On the other hand, in postnatal SMC-specific *Tgfbr2* knockout mice and LDS, the intact TGF-β receptor complexes are theoretically below one-fourth of the wild-type, which might be insufficient for the aortic wall maintenance and prevention of aortic aneurysm formation. In LDS, the resulting augmentation of TGF-β signaling via intact TGF-β receptor complexes would play active roles in disease progression, even though the molecular mechanisms of how TGF-β ligands are actively secreted and/or how inflammatory signals are upregulated remain to be determined. 

### 3.5. SMAD3 Gene Mutations Cause Loeys-Dietz Syndrome Type 3

*SMAD3* gene mutations were initially reported in patients presenting with aortic aneurysms and early-onset osteoarthritis (AOS), now referred to as LDS 3 with mild systemic features of MFS and LDS [76]. The heterozygous mutations lead to increased aortic expression of several key players in TGF-β signaling, including SMAD3.

Recently, van den Pluijm et al. reported that homozygous *Smad3* knockout mice (*Smad3^−/−^*) serve as a model for LDS 3 [92]. *Smad3^−/−^* mice develop aortic aneurysms rapidly and show intervertebral disc degradation and kyphosis [92,93]. Thus, phenotypic similarities between human LDS 3 and *Smad3^−/−^* mice were evident. However, there are some suspected differences between the two genetic conditions: both pSMAD2 and pERK1/2 were activated before aneurysms developed, however downstream TGF-β-activated target genes (*Fn-1, Pai-1*, and *Smad7*) were not upregulated in *Smad3^−/−^* aorta, reflecting the loss of a canonical signaling effector SMAD3. Especially, elastin disruption and pronounced pERK1/2 activation in the medial layer preceded aneurysmal formation that seemed to trigger adventitial inflammation.

### 3.6. TGFB2 and TGFB3 Mutations Cause Loeys-Dietz Syndrome Types 4 and 5

Mutations in genes encoding TGF-β ligands (*TGFB2* and *TGFB3*) cause familial TAADs, associated with mild systemic features of MFS and LDS [69,78,79], and now referred to as LDS 4 and LDS 5, respectively. The mutations are predicted to lead to loss of protein function; however, aortic tissues from affected patients showed increased TGF-β signaling cascades, in which the TGF-β ligand expression was instead normalized and/or upregulated [69,78,79].

Lindsay ME, et al. reported that heterozygous *Tgfb2* knockout mice (*Tgfb2^+/−^*) developed aortic aneurysms, suggesting that loss-of-function of a single allele of *Tgfb2* is sufficient to cause aortic aneurysms [69]. Importantly, there were no differences in either pSMAD2 or pERK1/2 in the dilated aorta at 4 months of age, compared to wild-type mice, whereas both pSMAD2 and pEAK1/2 activations were evident in the further dilated aorta at 8 months of age with increased TGF-β1 expression. In contrast, *Tgfb2^+/−^* mice crossed with *Fbn1^C1039G/+^* mice (*Tgfb2^+/−^*; *Fbn1^C1039G/+^*) showed a significant increase in aortic root dimension compared to either *Tgfb2^+/−^* or *Fbn1^C1039G/+^* mice at the early time points (2–4 months), associated with increased pSMAD2 activation.

These animal data described in the part of LDS 3–5 might also suggest the crucial roles of TGF-β signaling in the aortic wall maintenance, as discussed in LDS 1–2 and related genetic murine models (Figure 4C,D). Physiological levels of TGF-β signaling inhibits the aortic dilatation, and thus complete ablation of TGF-β signaling cascade may rapidly promote the aneurysmal formation. In contrast, the primarily and/or secondarily augmented TGF-β signaling in the heterozygous loss-of-function mutants would also exert to enhance the aortic dilatation. However, the differences in molecular processes by which the various types of aortic aneurysms were caused, must be further investigated and discussed.

## 4. Shprintzen-Goldberg Syndrome

Mutations in the *SKI* gene encoding the SKI proto-oncogene protein cause SGS, which is characterized by severe marfanoid habitus, camptodactyly, typical facial dysmorphism, craniosynostosis, and mild/moderate intellectual disability [94,95]. SKI exerts a negative regulatory effect on TGF-β signaling by interacting with other cellular partners, such as SMAD proteins and transcriptional co-regulators, including the nuclear receptor corepressors (N-CoRs), mSin3A, and histone deacetylases [94,96,97]. All pathogenic variants are localized in exon 1 and cluster into 2 domains: the R-SMAD binding domain and Dachshund homology domain (DHD), which mediates binding to SKI-interacting protein SKIP (SNW1) and N-CoR proteins.

The aortic aneurysm phenotype is usually less severe and penetrant in SGS patients, compared with MFS and LDS [95], and thus actual TGF-β signaling in the SGS aorta has not been properly analyzed. However, both canonical and noncanonical TGF-β signaling increase in primary dermal fibroblasts from SGS patients, and SKI is expressed in the developing and adult aorta. *Ski* morphant zebrafish closely recapitulate the human SGS craniofacial phenotype, with severe cardiac anomalies, including partial-to-complete failure in cardiac looping and malformations of the outflow tract [94]. These data indicate that SKI plays crucial roles in aortic morphogenesis and homeostasis, and aortic aneurysmal formation in SGS patients is a result of increased TGF-β signaling, similar to aortopathies of MFS and LDS.

## 5. Conclusions and Perspectives

Recent surgical treatments for MFS patients with ascending aortic aneurysms and dissections have improved their average life expectancy from 30 years to >70 years. However, a comprehensive understanding of the biology underlying multisystem disorders in MFS-related disorders remains insufficient. Genetic tests enabled the definite and rapid diagnosis, which might be useful for detection of high-risk subgroups requiring rapid surgical intervention, such as MFS patients with HI-type mutations and LDS patients. However, to date, there is not a genotype-specific medical treatment. The observed increased life expectancy did not come with an increased quality of life for middle-aged and elderly patients undergoing ARR, because these patients often progressively develop multiple aortic dilatations and dissections and kyphoscoliosis. Therefore, it is important to further investigate the target molecules and mechanisms underlying the development and progression of MFS-related disorders to provide more effective therapeutic strategies. In this review, we described some of the long-standing unsolved problems, such as the genotype-phenotype relationships and the TGF-β paradox. The molecular mechanisms for dilated cardiomyopathy (DCM)-like features (mild but significant left ventricular dilatation with impairment of left ventricular systolic function) [98,99,100] and pregnancy-related acute aortic dissection [101,102,103,104] also have been actively investigated and discussed elsewhere. To develop new treatment possibilities for MFS and related disorders, further large prospective clinical cohort studies and experimental studies are critical for validating the genotype-phenotype relationships and identifying confounding variables that influence various clinical phenotypes, including myocardial function, as well as potential differences in drug response [105]. In the near future, induced pluripotent stem (iPS) cell technology may provide key insights into the molecular mechanism and facilitate drug discovery also in this field [106,107].
